# Sexually dimorphic facial features vary according to level of autistic-like traits in the general population

**DOI:** 10.1186/s11689-015-9109-6

**Published:** 2015-04-15

**Authors:** Syed Zulqarnain Gilani, Diana Weiting Tan, Suzanna N Russell-Smith, Murray T Maybery, Ajmal Mian, Peter R Eastwood, Faisal Shafait, Mithran Goonewardene, Andrew JO Whitehouse

**Affiliations:** School of Computer Science and Software Engineering, University of Western Australia, 35 Stirling Highway, Crawley, 6009 Perth, WA Australia; Neurocognitive Development Unit, School of Psychology, University of Western Australia, 35 Stirling Highway, Crawley, 6009 Perth, WA Australia; School of Anatomy, Physiology and Human Biology, University of Western Australia, 35 Stirling Highway, Crawley, 6009 Perth, WA Australia; School of Dentistry/Oral Health Centre of Western Australia, University of Western Australia, 35 Stirling Highway, Crawley, 6009 Perth, WA Australia; Telethon Kids Institute, University of Western Australia, 100 Roberts Road, Subiaco, 6008 Perth, WA Australia

**Keywords:** Autism, Autism spectrum disorder, Hypermasculinisation, Gender defiant disorder, Facial features, Masculinity, Femininity, Raine study

## Abstract

**Background:**

In a recent study, Bejerot *et al*. observed that several physical features (including faces) of individuals with an autism spectrum disorder (ASD) were more androgynous than those of their typically developed counterparts, suggesting that ASD may be understood as a ‘gender defiant’ disorder. These findings are difficult to reconcile with the hypermasculinisation account, which proposes that ASD may be an exaggerated form of cognitive and biological masculinity. The current study extended these data by first identifying six facial features that best distinguished males and females from the general population and then examining these features in typically developing groups selected for high and low levels of autistic-like traits.

**Methods:**

In study 1, three-dimensional (3D) facial images were collected from 208 young adult males and females recruited from the general population. Twenty-three facial distances were measured from these images and a gender classification and scoring algorithm was employed to identify a set of six facial features that most effectively distinguished male from female faces. In study 2, measurements of these six features were compared for groups of young adults selected for high (*n* = 46) or low (*n* = 66) levels of autistic-like traits.

**Results:**

For each sex, four of the six sexually dimorphic facial distances significantly differentiated participants with high levels of autistic-like traits from those with low trait levels. All four features were less masculinised for high-trait males compared to low-trait males. Three of four features were less feminised for high-trait females compared to low-trait females. One feature was, however, not consistent with the general pattern of findings and was more feminised among females who reported more autistic-like traits. Based on the four significantly different facial distances for each sex, discriminant function analysis correctly classified 89.7% of the males and 88.9% of the females into their respective high- and low-trait groups.

**Conclusions:**

The current data provide support for Bejerot *et al*.’s androgyny account since males and females with high levels of autistic-like traits generally showed less sex-typical facial features than individuals with low levels of autistic-like traits.

## Background

A set of distinct facial features characterises a number of neurodevelopmental disorders such as Down syndrome (small and flat nose, small mouth, [1]) and Williams syndrome (flat nose, wide mouth and broad forehead, [2]). Autism spectrum disorder (ASD) is a neurodevelopmental disorder characterised by impairments in social and communication abilities and patterns of repetitive and stereotypical behaviours [3]. However, while ASD has not traditionally been thought to be characterised by distinctive facial features, this belief has been challenged in recent years.

In an early study of face morphology, Hammond *et al*. [4] reported that boys with ASD presented with greater facial asymmetry compared to controls. This finding led to the suggestion that facial information may be crucial in signalling aberrant brain development. Based on previous reports indicating that the brain and face of an individual arise from the same ectoderm layer in the embryo [5,6], Aldridge *et al*. [7] hypothesised that facial structures might reflect atypical neural development among individuals with ASD. Aldridge *et al*. [7] reported a distinct facial phenotype among 64 boys with ASD when compared to 41 typically developing boys. Some facial features that distinguished the two groups were distances between inner and outer corners of the eyes (increased in ASD), breadth of mouth (increased) and length of upper face (increased).

The largest study of the facial features of people with ASD was conducted by Ozgen *et al*. [8] as part of physical examinations of 112 children with ASD (93 boys and 19 girls) and 112 typically developing children matched on age and sex. There was a strikingly high prevalence of one or more major morphological anomalies (for example, open mouth appearance) among children with ASD (ASD: 43.8% *vs*. controls: 12.5%), and a greater proportion of children with ASD with one or more minor anomalies (for example, forehead prominence and face asymmetry; ASD: 98.2% *vs*. controls: 58.9%). Most notably, facial structure was the most prominent area of morphological abnormalities, though there appeared to be no consistent pattern of differences (for example, both unusually smaller and unusually larger mouth sizes were significantly more frequent in children with ASD compared to controls). Additionally, Ozgen *et al*. [8] reported that morphological abnormalities were more common in males with ASD compared to females with ASD. Therefore, the study provided some indication of sex differences in the prevalence of morphological abnormalities though one has to be cautious given the limited size of the female samples.

ASD is more prevalent in males compared to females, with a male-to-female ratio of approximately 4:1 [9]. The hypermasculinisation hypothesis attempts to account for this large gender disparity by suggesting that ASD is an extreme variant of male behaviour and cognition [10-12]. Under one instantiation of the hypothesis, the hypermasculinisation associated with ASD is said to be caused by the exposure to increased levels of prenatal testosterone *in utero*. Several studies have indicated that a higher level of prenatal testosterone (measured in amniotic fluid) is associated with more autistic-like traits reported by parents [13], less eye contact [14], poorer emotion recognition [15] and better performance in male-favouring cognitive tasks such as mental rotation [16] and disembedding [17]. Furthermore, lower second-to-fourth digit ratio, which is indicative of exposure to higher levels of prenatal testosterone [18], has been observed in individuals with ASD compared to typically developing controls [19,20]. Additionally, some studies have also found increased levels of postnatal testosterone among individuals with ASD relative to controls. Schmidtova *et al*. [21] reported increased levels of salivary testosterone among prepubertal (4 to 10 years old) and pubertal (11 to 18 years old) boys with ASD relative to comparison groups. In adults, Ruta *et al*. [22] reported elevated levels of androgens in the blood serum of men and women with ASD compared to typical controls. Given that both prenatal [23,24] and postnatal [23,25] testosterone levels are related to masculine facial features, one would expect individuals with ASD to present with more masculinised features compared to neurotypical individuals.

However, a recent study of the physical features of people with ASD provided data that challenge the hypermasculinisation hypothesis. Bejerot *et al*. [26] undertook several physical measurements (for example, waist-to-hip ratio, second-to-fourth digit ratio, head circumference and ankle circumference) of 50 adults diagnosed with ASD and 53 age-matched typically developed individuals, and also collected subjective ratings of photographs of their faces, voices and bodies using a ‘gender coherence’ scale. Females with ASD were found to have a less feminine pattern of physical features than typically developed females in terms of waist-to-hip ratio (higher), head circumference (larger) and ankle circumference (larger), as well as receiving lower ‘gender coherence’ ratings of their faces. In contrast, males with ASD showed a less masculine pattern of results than typically developed males on digit ratios (higher) and also received lower ‘gender coherence’ ratings of their bodies and voices. Based on these findings, Bejerot *et al*. [26] proposed that rather than be identified with hypermasculinisation, characteristics of ASD may be better conceptualised as being androgynous.

Taken together, there is now evidence that individuals with ASD possess a set of facial features that are distinct from the features of typically developing individuals [4,7,8]. However, the relationship between facial structure and autistic traits has yet to be explored with reference to masculinity and femininity using objective facial measurements. Such an investigation would provide novel evidence pertinent to the hypermasculinisation and androgyny hypotheses.

There is now wide agreement that autistic-like traits form a continuum in the general population with ASD representing the extreme end of the distribution [27,28]. To capitalise on this, we recruited adults from the general population selected for high and low levels of autistic-like traits and focused on differences in sexually dimorphic facial features in the two groups. There were two phases to our investigations. The aim of the first study was to establish, using a gender classification and scoring algorithm [29], the facial features that best differentiate samples of males and females drawn unselectively from the general population. Since facial structure changes with age [30] and ethnicity [31], the first study was conducted to identify the most sexually dimorphic facial features using participants of very similar age and background to the participants to be recruited for the second study. Recruitment for the second study then comprised screening young adults using the Autism-spectrum Quotient (AQ) questionnaire [32] to select groups of high and low AQ scorers for each sex. Critical interest then centred on whether the sexually dimorphic facial features identified in study 1 differentiated the groups with high *versus* low levels of autistic-like traits for each sex.

The hypermasculinisation and androgyny hypotheses can be used to generate competing predictions for the current study. Based on hypermasculinisation, it would be expected that individuals with high levels of autistic-like traits would display more masculinised facial features for males and less feminised features for females than their same-sex counterparts with low levels of autistic-like traits. Conversely, based on the androgyny account and findings reported by Bejerot *et al*. [26], it would be predicted that men with high levels of autistic-like traits would display less masculine facial features than men with low levels of those traits, while women with high levels of autistic-like traits would display less feminine facial features than women with low levels of those traits.

## Study 1

The aim of this study was to identify a set of facial features that best distinguish young adult males and females, using a gender classification and scoring algorithm [29]. We used three-dimensional (3D) images for all facial measurements and analyses because these are less susceptible to effects of illumination and pose variations and are capable of capturing facial surface information unlike two-dimensional (2D) images [33]. Using 3D images and objective measurements of facial features, Burton *et al*. [34] found that nose protuberance was the best facial feature for differentiating male from female faces in samples aged 18 to 30 years. Other features include length of cheek and philtrum length. In a more recent study, Velemínská *et al*. [35] found that men (*M*_age_ = 21.1 years) had smaller distances between the eyes, more deeply-set eyes with respect to facial plane, larger noses and wider chins compared to women (*M*_age_ = 21.6 years). Similarly, among male and female adolescents aged 12 to 18 years old, Chakravarty *et al*. [36] found that male adolescents had larger facial distances in features such as forehead, chin jaw, nose length, nose protuberance and philtrum length when compared to female adolescents.

## Methods

### Participants

The 208 participants (107 males; 101 females; *M*_age_ = 22.81 years, SD_age_ = 0.63 years) were recruited from the Western Australian Pregnancy Cohort (Raine) Study [37], an ongoing population-based longitudinal study. To control for the effects of ethnicity on face structure [31], only Caucasians were recruited for the study. Ethics approval was obtained from the Human Research Ethics Committee at the University of Western Australia, and written informed consent was obtained from each participant.

### Apparatus

3D images of the face were acquired using a 3dMDface system (3dMD, Atlanta, GA, USA) operated from a desktop computer in a room where lighting was kept constant. The 3dMDface system is a non-invasive imaging technology that produces 3D images (180° ear-to-ear frontal view) using random light projection on the face of the participant, as well as combining multiple 2D images captured using colour and infrared cameras from two stereo camera viewpoints. The use of infrared cameras allows the distance between the system and the participant to be standardised for all images obtained. Previous studies have shown that facial analyses using images captured by a 3dMDface system are highly precise and replicable [38,39]. A more detailed description of the 3dMDface system is available from http://3dMD.com/3dMDface/.

### Procedure

Participants sat in front of the 3dMDface scanner with the distance between the chair and scanner adjusted so their faces appeared in the middle of the computer screen. During the imaging process, participants fixated their gaze on a sticker pasted on the wall behind the scanner, maintained a neutral facial expression and kept their mouths closed. No accessories were worn for the imaging process and loose hair was pinned back from the face.

### Gender classification and scoring algorithm

The algorithm is capable of identifying sexually dimorphic facial features (classification accuracy of 94%) and assigning gender scores to 3D face images using objective measurement. The algorithm is briefly summarised in this paper and further details can be found in [29].

**Figure 1:**
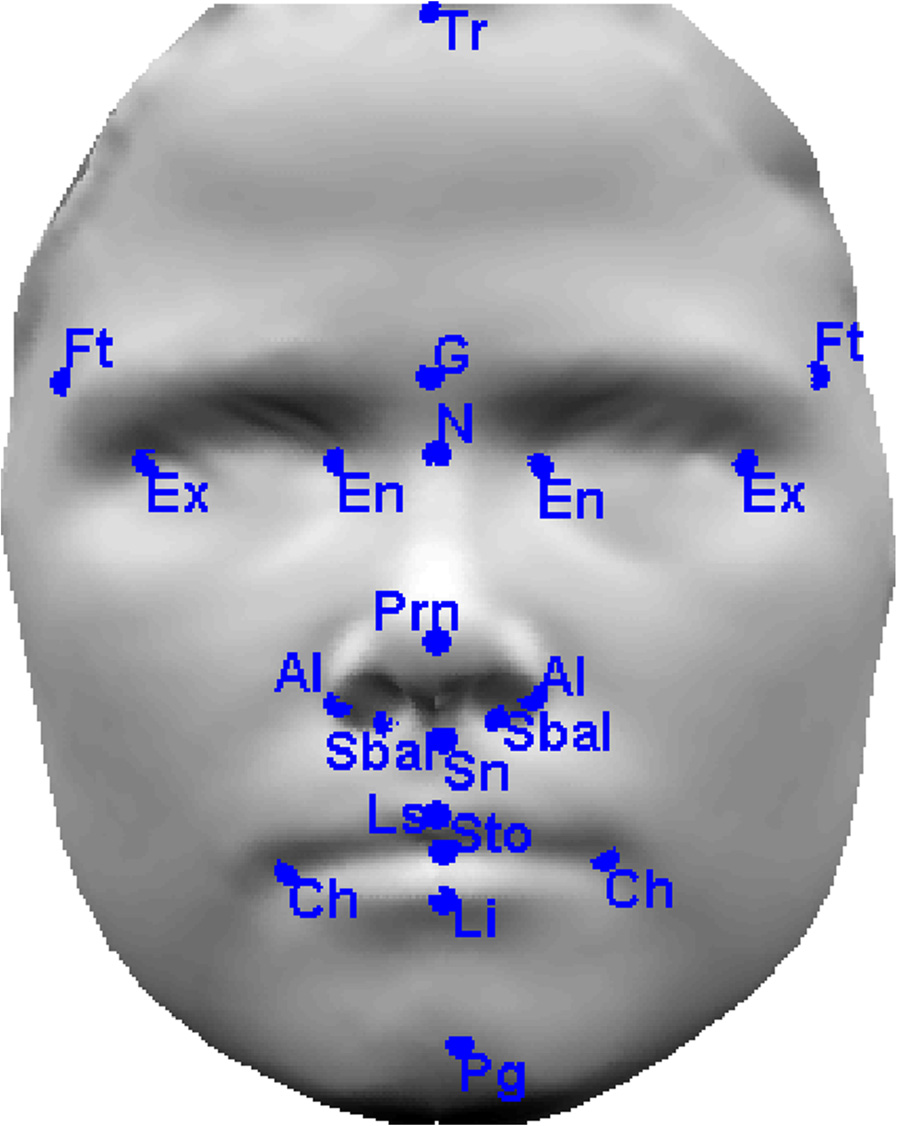
3D image annotated with 21 facial landmarks.

In the first stage of the analysis, 21 facial landmarks, defined by Farkas [40], were annotated on each image (see Figure 1) and 23 linear distances (see Table 1) were measured using Matlab. Next, the minimal redundancy maximal relevance algorithm was used to select features that were most relevant for distinguishing male and female faces. The final stage involved training a linear discriminant analysis (LDA) classifier [41] using the selected features. The training was performed using a tenfold validation technique in which nine folds were used for training the classifier and one fold for testing. The LDA classifier identified an optimal subset of facial features that most effectively and accurately classifies the 3D face images into their respective gender.

**Table 1:** **Summary of facial landmarks and distances measured in study 1**

**Number**	**Landmark**	**Facial distance**
1	*Ft*-*Ft*	Forehead width
2	*Ex*-*Ex*	Outer canthal width
3	*N*-*Prn*	Nasal bridge length
4	*Sn*-*Prn*	Nasal tip protrusion
5	*Sn*-*Ls*	Philtrum length
6	*Al*-*Al*	Nose width
7	*Ex-En (left)*	Eye fissure length (left)
8	*Ex-En (right)*	Eye fissure length (right)
9	*En-En*	Intercanthal width
10	*Ch-Ch*	Mouth width
11	*N-Sto*	Upper facial height
12	*N-Sn*	Nose height
13	*En-N (left)*	Nasal root height (left)
14	*En-N (right)*	Nasal root height (right)
15	*Sn-Sto*	Upper lip height
16	*Ls-Sto*	Upper vermillion height
17	*Sto-Li*	Lower vermillion height
18	*Tr-G*	Forehead height
19	*Sto-Pg*	Mandible height
20	*Sbal-Sbal*	Alar base width
21	*Sbal-Prn*	Alar length
22	*Tr-Prn*	Upper profile height
23	*Prn-Pg*	Lower profile height

### Statistical analysis

Independent-samples *t*-tests were conducted to confirm that the linear distance for each of the selected facial features differed significantly between males and females.

## Results

From the 23 linear distances measured, LDA identified a subset of six distances that provided the highest sex classification accuracy (97.19% for males and 95.04% for females). These features were forehead width (*Ft*-*Ft*), outer canthal width (*Ex*-*Ex*), nasal bridge length (*N*-*Prn*), nasal tip protrusion (*Sn*-*Prn*), philtrum length (*Sn*-*Ls*) and nose width (*Al*-*Al*).

Independent-samples *t*-tests revealed that all six of these sexually dimorphic facial features were significantly larger in distance in males than in females (see Table 2): forehead width, *t*(206) = 14.30, *P* < .001, *r*^2^ = .50; outer canthal width, *t*(206) = 3.75, *P* < .001, *r*^2^ = .06; nasal bridge length, *t*(206) = 10.18, *P* < .001, *r*^2^ = .33; nasal tip protrusion, *t*(206) = 9.24, *P* <.001, *r*^2^ = .29; philtrum length, *t*(206) = 3.02, *P* = .003, *r*^2^ = .04; nose width, *t*(206) = 10.59, *P* < .001, *r*^2^ = .35.

**Table 2:** **Means (and standard deviations) in millimetres of the critical facial distances for each sex**

	**Male (** ***n*** **= 107)**	**Female (** ***n*** **= 101)**	***P***
*Ft*-*Ft*: forehead width	124.12 (5.27)	113.41 (5.53)	<.001
*Ex*-*Ex*: outer canthal width	96.57 (4.54)	94.29 (4.20)	<.001
*N*-*Prn*: nasal bridge length	47.34 (3.77)	42.12 (3.62)	<.001
*Sn*-*Prn*: nasal tip protrusion	19.67 (1.95)	17.18 (1.94)	<.001
*Sn*-*Ls*: philtrum length	8.17 (2.44)	7.25 (1.94)	.003
*Al*-*Al*: nose width	32.71 (2.58)	29.08 (2.35)	<.001

## Study 2

Study 2 aimed to examine whether the six sexually dimorphic facial features identified in study 1 could distinguish, for each sex, faces of groups of individuals selected for high and low levels of autistic-like traits.

## Methods

### Participants

Based on the lower and upper quartiles of the score distribution of the Autism-spectrum Quotient (AQ; [32]), the cut-off scores for low and high scores were ≤10 and ≥22, respectively. These cut-off scores are comparable to those employed in previous studies reporting differences between high and low AQ groups (see, for example, [42]), and are extreme with reference to the nonclinical samples reviewed by Ruzich *et al*.[43], which yielded a weighted mean AQ of 16.94% and a 95% confidence interval of 11.6 to 20.0 for the 78 samples. Sixty-one (38 males) of the Raine participants (described in study 1) met the criterion for either high- or low-AQ group inclusion. These volunteers had completed the AQ in an earlier study [44] (mean age at time of testing = 19.74 years, SD = 0.79 years). An additional 51 Caucasian undergraduates (20 males; 31 females; *M*_age_ = 19.0 years, SD = 1.94 years) who also met inclusion criteria were recruited from the University of Western Australia to increase sample sizes. The final sample comprised 112 young adults (58 males), and descriptive statistics are provided in Table 3.

**Table 3:** **Descriptive statistics and critical facial distances for the pairs of high- and low-AQ groups for each sex**

	**Males**			**Females**		
	**Low-AQ**	**High-AQ**	***P***	**Low-AQ**	**High-AQ**	***P***
	**(** ***n*** **= 33)**	**(** ***n*** **= 25)**		**(** ***n*** **= 33)**	**(** ***n*** **= 21)**	
AQ score						
M (SD)	8.82 (1.04)	25.20 (2.26)		6.58 (1.60)	26.48 (3.63)	
Range	7 to 10	22 to 30		1 to 8	22 to 35	
Age						
M (SD)	19.24 (1.20)	19.72 (2.01)		18.91 (1.63)	19.29 (1.15)	
Range	17 to 22	17 to 26		17 to 23	17 to 22	
Facial distances (mm)						
Forehead width						
M (SD)	126.01 (5.74)	121.11 (5.69)	.002	110.63 (5.11)	116.53 (4.73)	<.001
Outer canthal width						
M (SD)	98.38 (4.38)	94.26 (4.47)	.001	90.95 (3.61)	96.59 (4.44)	<.001
Nasal bridge length						
M (SD)	49.37 (4.38)	46.17 (3.69)	.005	43.83 (3.00)	40.40 (3.64)	<.001
Nasal tip protrusion						
M (SD)	21.62 (1.94)	18.65 (2.29)	<.001	19.52 (2.10)	19.90 (2.31)	.538
Philtrum length						
M (SD)	22.99 (2.17)	23.67 (3.05)	.323	21.25 (2.49)	21.62 (2.58)	.601
Nose width						
M (SD)	32.81 (3.10)	32.46 (2.42)	.642	28.58 (2.10)	30.38 (2.01)	.003
Facial area (mm^2^)						
M (SD)	24,600 (1,670)	24,500 (1,870)	.807	22,800 (1,620)	23,100 (1,650)	.569

M, mean; SD, standard deviation; AQ, Autism-spectrum Quotient.

### Autism-spectrum Quotient (AQ)

The AQ [32] is a self-report questionnaire that assesses levels of autistic-like traits in the general population. Participants are provided with 50 statements (for example, ‘People often tell me that I keep going on and on about the same thing.’ and ‘I am fascinated by numbers.’) and indicate how well each statement applies to them (score range: 0 to 50). The AQ has good test-retest reliability (*r* = .70, [32]) and its major factors (social skills, attention to patterns/details and communication: see [45]) correspond to key dimensions of ASD symptoms. The instrument reliably distinguishes ASD and neurotypical groups [32,46] and high-AQ samples differ from their low-AQ counterparts in ways that mirror ASD *versus* control differences (for example, [42,47,48]).

### Apparatus and procedure

The 3dMDface system and imaging procedure described in study 1 were also used in this study to obtain the 3D face images.

### Statistical analysis

We annotated 12 facial landmarks on each image and measured the six distances that were found to be sexually dimorphic in study 1. Independent-samples *t*-tests were conducted to compare the six distances between participants in the low- and high-AQ groups within each sex. Discriminant function analyses were then used to classify participants into their respective AQ groups based on the facial distances that were significantly different.

## Results

Descriptive statistics for the face measurements are shown in Table 3. Four of the six distances were found to be significantly different between high- and low-AQ groups within each sex. Generally, males in the high-AQ group were found to have less masculine features (smaller distances) than males in the low-AQ group: forehead width, *t*(56) = 3.24, *P* = .002, *r*^2^ = .16; outer canthal width, *t*(56) = 3.51, *P* = .001, *r*^2^ = .18; nasal bridge length, *t*(56) = 2.95, *P* = .005, *r*^2^ = .13; and nasal tip protrusion, *t*(56) = 5.34, *P* < .001, *r*^2^ = .34. Females in the high-AQ group were found to have less feminine features (larger distances) than their low-AQ counterparts for three of the six features: forehead width, *t*(52) = 4.26, *P* < .001, *r*^2^ = .26; outer canthal width, *t*(52) = 5.11, *P* < .001, *r*^2^ = .33; and nose width, *t*(52) = 3.12, *P* = .003, *r*^2^ = .16. The only significant distance to not fit this pattern was nasal bridge length, with this feature shorter in length (more feminine) in the high-AQ females compared to the low-AQ females, *t*(52) = 3.76, *P* < .001, *r*^2^ = .21.

Since it is possible that body mass index could affect facial distances, a secondary analysis investigated whether there were differences in total facial area between the high- and low-AQ groups. Area of the face was calculated using the 3D point cloud that defined each face and the triangular connectivity between these points. Independent samples *t*-tests identified no group differences in total facial area for males (*P* = .807) or females (*P* = .569; see Table 3). This indicates that differences in facial distances were not due to differences in overall face size.

For each sex, the four significantly different distances were entered into a discriminant function analysis aimed at classifying participants into their high- and low-AQ groups. In males, all four facial distances were significant predictors of group membership, *χ*^2^ = 40.7, *P* < .001 (see Table 4 for standardised coefficients). Classification results showed that 89.7% (cross-validated percentage of 84.5%) of the male participants were correctly classified into their respective AQ groups. In females, all four facial distances were also significant predictors, *χ*^2^ = 29.0, *P* < .001 (see Table 4) with 88.9% (cross-validated percentage of 79.6%) of the women correctly classified into their AQ groups.

**Table 4:** **Facial distances entered into discriminant function analysis for each sex**

	**Standardised coefficient**	
**Facial distances**	**Male**	**Female**
Forehead width	.41	.67
Outer canthal width	.44	.80
Nasal bridge length	.37	−.59
Nasal tip protrusion	.67	Not entered
Nose width	Not entered	.49

Standardised coefficients with an absolute value greater than .30 are considered significant predictors [69].

## Discussion

The current study used 3D technology and a gender classification and scoring algorithm [29] to investigate the facial phenotypes of males and females in the general population, as well as young adults selected for high and low levels of autistic-like traits. Study 1 aimed to identify facial features that distinguished the faces of males and females in the general population. LDA identified a set of six facial distances capable of classifying the young adult male and female faces with an accuracy of 97.19% and 95.04%, respectively. Several of the sexually dimorphic facial features found in the current study are consistent with previous findings, such as nasal tip protrusion [34], philtrum length [34,36] and the widths of forehead and nose [35,36].

Study 2 then examined these six sexually dimorphic facial features in groups of males and females selected for high and low levels of autistic-like traits. Among males, four of the six facial features (forehead width, outer canthal width, nasal bridge length and nasal tip protrusion) were significantly less masculinised in the high-AQ group compared to the low-AQ group. Among females, four features were significantly different between the high- and low-AQ groups. Three features (forehead width, outer canthal width and nose width) were less feminised in the high-AQ group compared to the low-AQ group while one feature (nasal bridge length) was more feminised in the high-AQ group. These findings indicate that individuals with high levels of autistic-like traits generally show less sex-typical facial characteristics than those with low levels of these traits. Intriguingly, however, the outcome for nasal bridge length among the females was in the opposite direction to the general pattern of results. Bruce *et al*. [49] argued that when observers classify faces as male or female, some of the features contributing to reliable classification, such as forehead width and nose protuberance, are processed locally (that is, independent of other features) while other features, such as nasal bridge length, are processed configurally (that is, relative to other features such as the width of the face). Although the current study examined objective measurements of faces rather than perceptual classification of sex, a possible extension of the current research would be to evaluate whether configurations of features that differentiate males and females assist in differentiating groups selected to differ in levels of autistic-like traits. The utility of ratios such as the ratio of nasal bridge length to width of the face would be of particular interest.

Results of the current study are generally consistent with Bejerot *et al*.’s [26] androgyny account and run counter to the hypermasculinisation hypothesis. In a recent study, Lai *et al*. [50] presented neuroanatomical data obtained from males and females with and without ASD. The neuroanatomy of ASD was found to be sex-dependent. More specifically, females with ASD showed a more ‘masculinised’ neuroanatomy compared to females without ASD. However, the brain masculinisation we would expect based on the hypermasculinisation account was not observed in males. Lai *et al*. made further investigations by comparing two sexually dimorphic brain structures that characterised ‘feminisation’ in males and found that one of the two brain areas showed more feminisation in males with ASD compared to those without ASD.

Brain masculinisation in females with ASD and feminisation in males with ASD reported by Lai *et al*. [50] is in line with the pattern of results reported in Bejerot *et al*. [26] and in the current study. Given that the development of the brain and face occurs in concert *in utero* [4], these results further strengthen the position that facial information may provide a crucial marker of aberrant neurodevelopment in ASD. In addition, these results also indicate that characteristics of ASD may manifest differently in males and females. While the hypermasculinisation hypothesis may account for masculinisation in females, the androgyny hypothesis may provide a more complete description of the characteristics of ASD in both sexes.

Nonetheless, some research outcomes have been consistent with the hypermasculinisation account and others not. Scott *et al*. [51] reported evidence of an association between higher AQ scores and more masculinised faces for males but not for females. While these results are difficult to reconcile with those observed in the current study, there are important methodological differences. For example, Scott *et al*. [51] obtained masculinity indices via subjective ratings, whereas the current study used objective markers of facial morphology. Furthermore, the face stimuli used by Scott *et al*. [51] were those of mid-AQ individuals that had been synthetically morphed towards composite facial averages derived from high- and low-AQ participant samples. By contrast, the current study examined objective markers in the actual faces of people with high- or low-AQ, thus providing a more direct test of the hypotheses.

More broadly, while there has been reports of prenatal testosterone concentrations being positively associated with the development of autistic-like traits [13] and ASD [52] later in life, there is no evidence of a link between perinatal testosterone (measured from cord blood) and autistic-like traits [44]. Regarding postnatal testosterone, some studies reported a positive association between testosterone levels and autistic-like traits [22,53], while one study found no such association [54]. In Bejerot *et al*. [26], females with ASD were found to have higher levels of testosterone compared to female controls, but no difference was found in males.

In addition, there have been numerous studies that have reported hypermasculinised cognitive abilities for both males and females with ASD. For instance, individuals with ASD tend to perform better on male-favouring tasks (for example, mental rotation [16]) and perform worse on female-favouring tasks (for example, emotion recognition [15]). Although these findings based on cognitive data are difficult to reconcile with the current findings based on physical characteristics, an early study by Petersen [55] reported data that provides a link between masculinised cognition and androgynous physical features. Using masculinity-femininity ratings of images of adolescent bodies, Petersen found that males with bodies rated as less masculine (more androgynous) performed better on a spatial task (a male-favouring ability) but worse on a verbal task (a female-favouring ability) relative to males with more masculine body ratings. As for female adolescents, those with less feminine bodies showed better performance on the spatial task but equivalent performance on the verbal task, relative to those with more feminine bodies. Thus, for both sexes, androgynous body ratings were associated with a pattern of cognitive performance typically associated with masculinisation.

At present, the specific biological mechanisms that could underlie the development of androgynous facial features and their relationships with autistic traits remain unclear. Embryonic craniofacial development is genetically predetermined and can also be influenced by extrinsic environmental factors [56]. Potential candidate genes have been identified among some individuals with ASD [57]. It is possible that these genes not only express themselves in the form of autistic traits but also influence facial phenotypes of those with ASD. In addition, it is possible that fetal environment factors such as levels of prenatal testosterone influence both neurodevelopment and face development in concert.

To our knowledge, this is the first study to explore the degree of facial masculinity and femininity using objective measurement in healthy individuals selected for high and low levels of autistic-like traits. The strength of the study is that participants in both phases of the study were matched on age and background to ensure validity in the facial features assessed. The current study also employed the use of advanced face scanning technology and software algorithms to obtain reliable objective measurements and analyses of facial features.

However, three features of the study constrain interpretation of the results. First, while the AQ is a valid and efficient instrument to use in screening large numbers of neurotypical participants to recruit groups with high and low levels of autistic-like traits, it does not assess clinically significant symptoms. Future research of this kind would benefit from checking for ASD symptoms using an instrument such as the Autism Diagnostic Observation Schedule, Second Edition [58] or Autism Diagnostic Interview-Revised [59]. Second, the current study did not investigate clinical samples of individuals diagnosed with ASD. However, population-based studies have provided support for a smooth etiological continuum of autistic-like traits across the general population, with clinical ASD representing the extreme end of a quantitative distribution [60]. The current findings warrant future research to examine if similar results can be replicated in comparing ASD and neurotypical samples. Lastly, with the current study investigated facial features for Caucasian participants, our findings cannot be generalised to other ethnic populations. Nevertheless, since some studies have shown that the sexual dimorphism of facial features is culturally stable [61,62] and numerous studies have found the AQ to be culturally stable [63-65], we would anticipate similar AQ group differences in face morphology for other ethnic populations as well. An extension of the current study would be to investigate other sexually dimorphic physical characteristics such as voice [66,67] and body composition [68] in relation to autistic-like traits to determine whether the androgyny account applies to these physical features as well.

## Conclusions

Bejerot *et al*. [26] provided preliminary evidence for the androgyny account using subjective ratings of faces of individuals with ASD and typically developed individuals. The findings of the current study extend the work of Bejerot *et al*. [26] by using objective measurements of facial features obtained from 3D images. Results of the current study provided further support for the androgyny account in which both males and females with high levels of autistic-like traits were found to typically present more androgynous (that is, less masculine for males and less feminine for females) facial features when compared to males and females with low levels of autistic-like traits.

## Abbreviations

2D: two-dimensional
3D: three-dimensional
AQ: Autism-spectrum Quotient
ASD: autism spectrum disorder
LDA: linear discriminant analysis
